# Intravascular ultrasound-guided percutaneous treatment of late-presenting left anterior descending artery compression after penetrating cardiac trauma in a young adult: a case report

**DOI:** 10.1093/ehjcr/ytag621

**Published:** 2026-08-30

**Authors:** Hatice Özdamar, Duygu Melisa Hökerek, Burçin Abud, Burak Ayça, Mehmet Emre Özpelit

**Affiliations:** Department of Cardiology, Tepecik Training and Research Hospital, Izmir 35020, Turkiye; Department of Cardiology, Tepecik Training and Research Hospital, Izmir 35020, Turkiye; Department of Cardiovascular Surgery, Tepecik Training and Research Hospital, Izmir 35020, Turkiye; Department of Cardiology, Buca Seyfidemirsoy State Hospital, Izmir 35390, Turkiye; Department of Cardiology, Medical Point University Hospital, Izmir 35575, Turkiye

**Keywords:** Case report, Penetrating cardiac trauma, Iatrogenic coronary stenosis, Left anterior descending artery, Intravascular ultrasound, Multimodal imaging

## Abstract

**Background:**

Penetrating cardiac trauma is rare in adolescents, and late acute coronary syndrome after surgical repair, which narrows left anterior descending artery, is even rarer. Managing ischaemia from late coronary stenosis without underlying atherosclerosis is challenging. Multimodal imaging, including coronary computed tomography and intracoronary imaging, is crucial for guiding treatment.

**Case summary:**

An 18-year-old male presented with recurrent chest pain. Two years after surgical repair of a penetrating left ventricular injury, a 12-lead electrocardiogram showed deep, symmetrical T-wave inversions in the anterolateral leads. Coronary angiography revealed a myocardial bridge and a significant stenosis in the mid left anterior descending artery (LAD). After recurrent symptoms, coronary computed tomography angiography demonstrated repair material surrounding the stenotic segment. Myocardial perfusion imaging showed a small fixed defect without reported reversibility. Intravascular ultrasound (IVUS) showed a minimum lumen area of 1.85 mm^2^ without an atherosclerotic phenotype. Following individualized Heart Team review favoring a percutaneous approach, a drug-eluting stent was implanted; the final minimum stent area was 4.83 mm^2^. At 1 month, exertional chest pain had markedly improved, biomarkers were normal, and the electrocardiogram was unchanged.

**Discussion:**

This case may represent one of the initial documented instances of IVUS-guided percutaneous coronary intervention (PCI) for late LAD compression caused by mechanical factors, following pledget repair for penetrating cardiac trauma in a young patient. Multimodal imaging helped clarify the underlying mechanism of this non-atherosclerotic coronary lesion, and a multidisciplinary Heart Team approach directed the personalized treatment decision.

Learning points

- In young patients with a history of cardiac surgery, particularly pledgeted repair, consider late iatrogenic coronary compression if ischaemic symptoms recur.
- Multimodal imaging helps differentiate mechanical compression from atherosclerosis; IVUS-guided PCI can be an option when surgery is not suitable, but it requires long-term monitoring.

## Introduction

Penetrating cardiac trauma in adolescents is uncommon, and mostly results from stab wounds.^1^ Associated coronary injuries are usually managed with emergency coronary artery bypass grafting within the first few hours.^2^ In patients with isolated left ventricular injury, surgical repair with pledget-reinforced sutures or Bioglue is lifesaving, but late coronary complications from the repair itself are rare and poorly understood. In the absence of atherosclerosis, standard risk-assessment tools do not apply.

Iatrogenic coronary artery compression following cardiac surgery has been documented, often related to prosthetic valve deformation and pericardial patch repair.^3^ Additionally, late coronary stenosis due to pledget material after repair of penetrating cardiac trauma has been reported previously.^4,5^ However, to our knowledge, intravascular ultrasound (IVUS)-guided percutaneous coronary intervention (PCI) for a symptomatic, mechanically induced coronary lesion in this specific scenario has not been described before. We present an 18-year-old male who developed late iatrogenic stenosis of left anterior descending artery (LAD), caused by Teflon felt pledgets used during left ventricular repair 2 years earlier, managed with IVUS-guided PCI after comprehensive multimodal imaging and Heart team assessment.

## Summary figure

**Figure:**
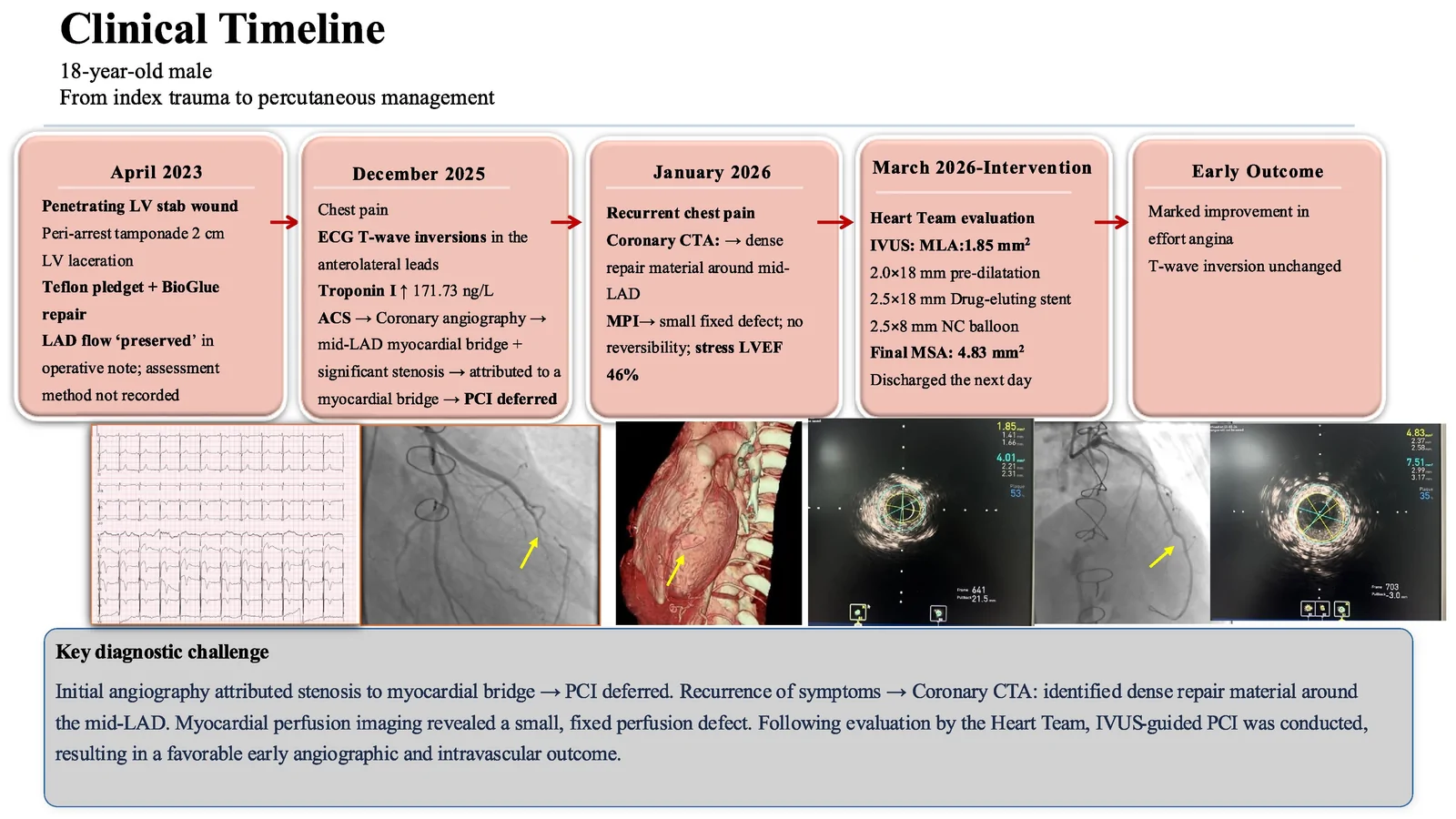


## Case presentation

**Figure 1:**
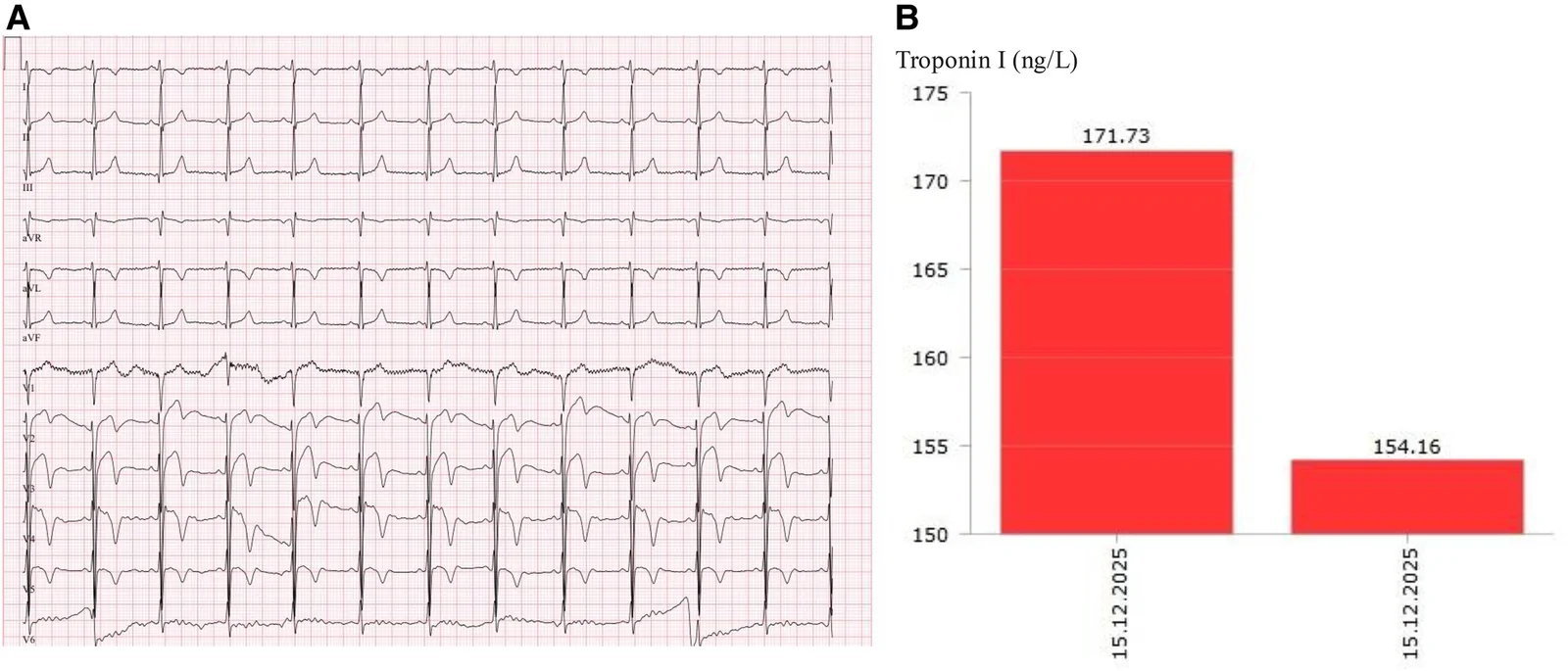
First episode of chest pain—initial workup. *(A)* Electrocardiogram demonstrating deep, symmetrical T-wave inversions in the anterolateral leads. *(B)* Troponin I was initially 171.73 ng/l (normal value <46.0 ng/l); on repeat testing, it had decreased to 154.16 ng/l.

**Figure 2:**
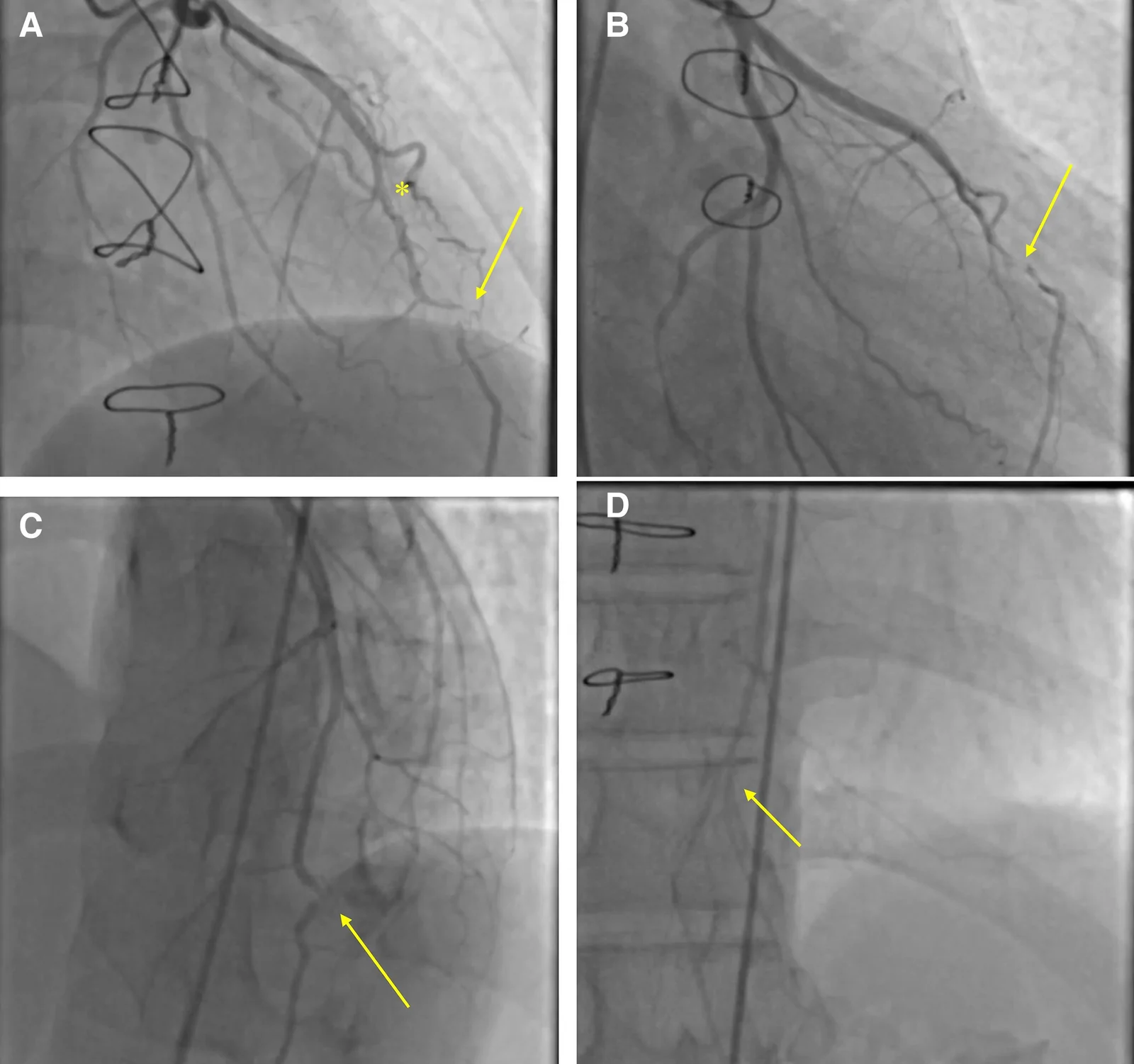
Diagnostic coronary angiography of the left coronary system and left internal mammary artery. (*A*) Anteroposterior view: systolic compression and a milking effect distal to the diagonal branch (asterisk), with a high-grade stenosis pronounced during systole (arrow). (*B*) Right anterior oblique view: a high-grade luminal narrowing is observed distal to the diagonal branch. *(C)* Left anterior oblique cranial view: during diastole, a normal left anterior descending artery segment distal to the diagonal branch is seen, followed by an anomalous course in the mid- left anterior descending artery (arrow). (*D*) Left internal mammary artery angiography showing a diminutive and distally attenuated left internal mammary artery.

An 18-year-old male with a history of penetrating cardiac injury and left ventricular repair 2 years earlier arrived at a state hospital complaining of chest pain. His blood pressure was 106/71 mmHg, and his heart rate was 83 beats/min; the physical examination was otherwise unremarkable. ECG showed deep, symmetrical anterolateral T-wave inversion (*Figure 1A*); troponin I was 171.73 ng/l (reference <46.0 ng/l), decreasing to 154.16 ng/l on repeat testing (*Figure 1B*). Given a working diagnosis of acute coronary syndrome, coronary angiography showed normal right coronary and circumflex arteries, a myocardial bridge in the mid-to-distal left anterior descending artery (LAD) with a stenosis pronounced during systole, and a diminutive (*Figure 2A–C*, see Supplementary material online, *Video S1*), distally attenuated LIMA (*Figure 2D*, see Supplementary material online, *Video S1*). The stenosis was attributed to the bridge; PCI was deferred, and the patient was discharged on medical therapy with instructions to seek tertiary centre care if angina recurred.

Two years prior, the details of the index surgery, including treatment for cardiac tamponade and a 2 cm left ventricular laceration (AAST-OIS Grade V), repair with Teflon felt pledgets and BioGlue, and maintenance of intraoperative LAD flow, were available only from the operative report, as no direct information from the surgical team was available.

**Figure 3:**
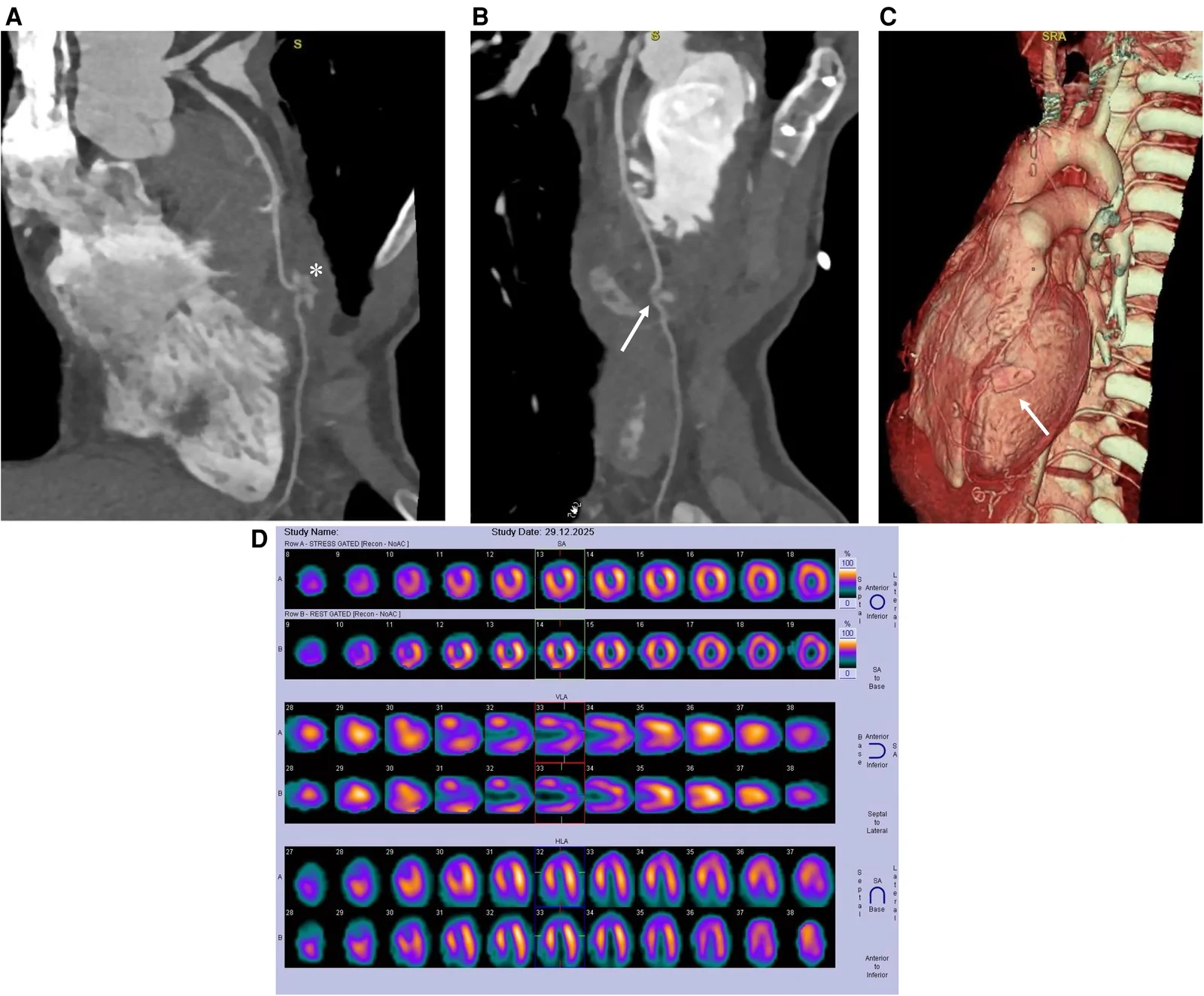
Anatomical and functional assessment. *(A)* Multiplanar reconstruction images showing that the abnormality in the mid-left anterior descending artery corresponds to pledget material concentrically surrounding this segment (asterisk). *(B)* Extrinsic compression by the pledget material on the coronary vessel wall results in high-grade luminal narrowing of the mid-left anterior descending artery, present even during diastole, consistent with a structurally fixed, non-dynamic stenosis. *(C)* Three-dimensional volume rendering illustrating the extrinsic compression exerted by the pledget material (arrow). *(D)* Myocardial perfusion imaging revealed a small, fixed perfusion defect in the anteroseptal, apical, and apico-medial segments.

One month later, the patient presented to our tertiary centre with normal troponin and NT-proBNP but unchanged, persistent T-wave inversion; echocardiography showed preserved LV function. A chronic coronary syndrome was suspected, and the outside angiogram was retrospectively reviewed. Coronary computed tomography angiography demonstrated a short concentric stenosis distal to the bridge and dense repair material surrounding the involved LAD segment, strongly suggesting a surgical relationship (*Figure 3A–C*). A 2-day adenosine (140 µg/kg/min) stress/rest technetium-99m sestamibi study showed a small low-to-moderate-intensity fixed anteroseptal, apical, and apico-medial defect, minimal regional hypokinesia, and stress left-ventricular ejection fraction of 46% (*Figure 3D*); no reversibility was reported. Summed scores involved myocardial percentage, transient ischaemic dilatation, and rest ejection fraction were unavailable.

**Figure 4:**
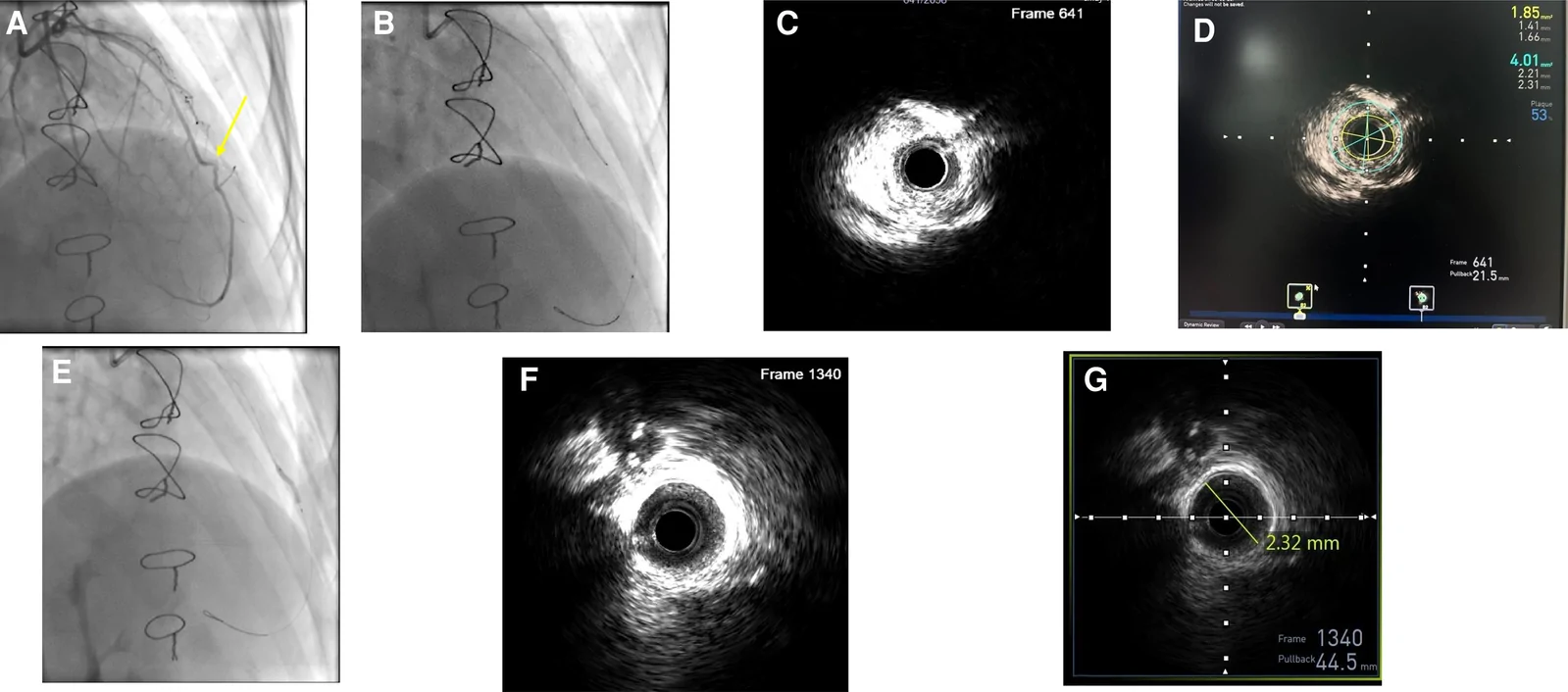
Pre-percutaneous coronary intervention coronary angiography and intravascular ultrasound assessment of the left anterior descending artery lesion. *(A)* Right anterior oblique cranial view of coronary angiography showing the mid-left anterior descending artery lesion (arrow). *(B)* Insertion of the intravascular ultrasound (Opticross) 3F probe into the target lesion of the left anterior descending artery. *(C)* Intravascular ultrasound imaging reveals the most severe stenosis, with a concentric, fibrotic appearance. *(D)* Intravascular ultrasound measurement of the minimum lumen area, 1.85 mm^2^, without an atherosclerotic phenotype. *(E)* Pre-dilation of the left anterior descending artery lesion was performed using a semi-compliant Shun Loach PTCA balloon, 2.0 × 18 mm (Shunmei Medical Co., China). *(**F**, **G**)* Immediately after pre-dilation, intravascular ultrasound showed a vessel diameter of 2.32 mm at the treated segment.

IVUS-guided diagnostic coronary angiography was planned (*Figure 4A–C*). IVUS confirmed a concentric, fibrotic, non-atherosclerotic stenosis (minimum lumen area 1.85 mm^2^ of a 4.01 mm^2^ reference; plaque burden 53%) (*Figure 4D*, see Supplementary material online, *Video S2*). Invasive physiological assessment was not performed, as the lesion was morphologically severe on IVUS and its immediate proximity to the myocardial bridge could confound standard FFR/iFR interpretation, given the bridge's own dynamic effect on coronary flow. Cardiac surgery was consulted regarding bypass; redo surgery risked disrupting the ventricular repair in an adhesed field, while the LIMA was diminutive and attenuated distally. The Heart Team elected percutaneous management.

**Figure 5:**
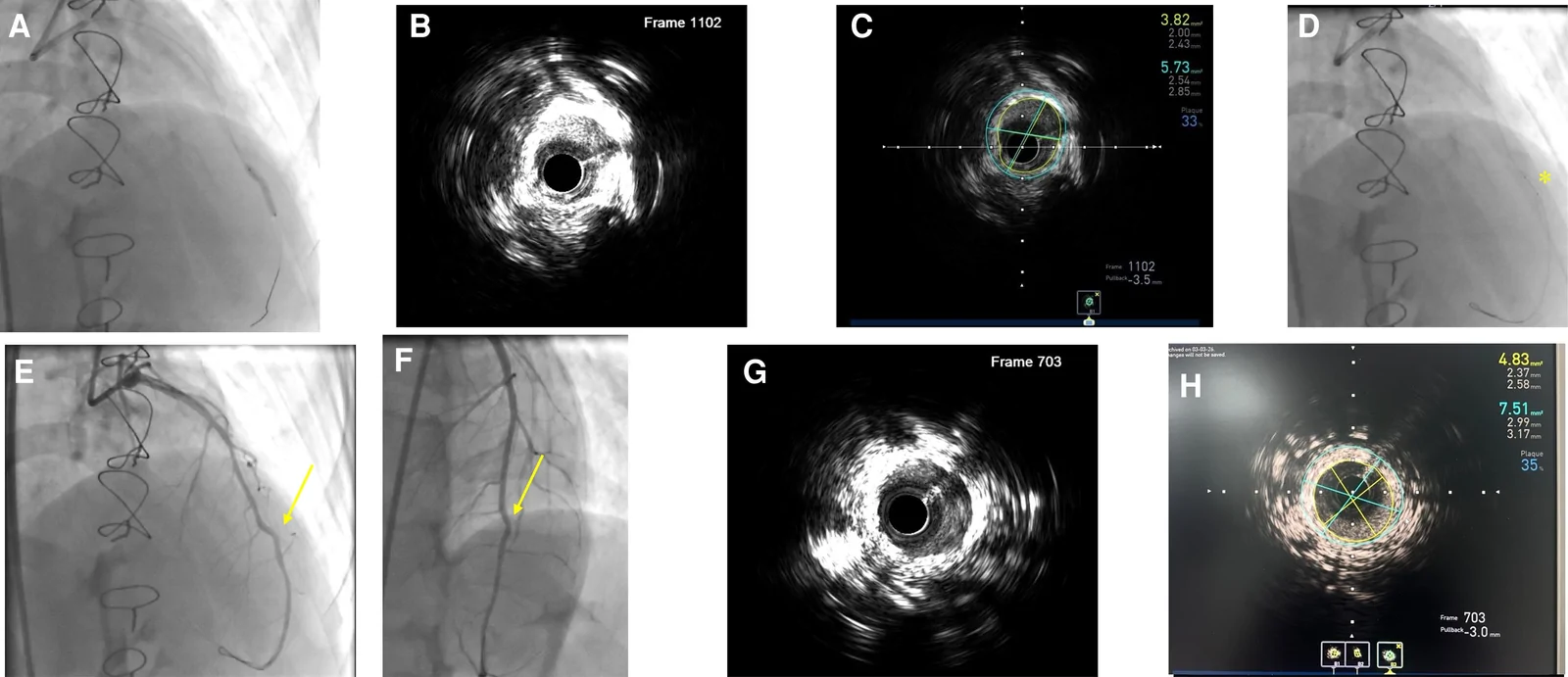
Post-percutaneous coronary intervention coronary angiography and intravascular ultrasound assessment of the left anterior descending artery lesion. *(A)* Deployment of a Xience Pro™ drug-eluting stent (2.50 × 18 mm, Abbott, USA) in the mid-left anterior descending artery. *(B)* Intravascular ultrasound was used to verify stent position and expansion after deployment. *(C)* Intravascular ultrasound shows residual stent underexpansion; the minimum stent area was estimated at 3.82 mm^2^ (reference area 5.73 mm^2^; plaque burden 33%). *(D)* A 2.5×8 mm non-compliant Solaris balloon (asterisk) (Medtronic, MN, USA) was used at high pressure (16 atmospheres). *(E, F)* Right anterior oblique cranial and left anterior oblique cranial views of the final coronary angiogram show a favourable angiographic result (arrow). *(G)* Repeat intravascular ultrasound shows full lesion coverage by the drug-eluting stent. *(H)* Final intravascular ultrasound measurement of the minimum stent area was 4.83 mm^2^ (reference area 7.51 mm^2^; plaque burden 35%; ∼64% of the reference lumen area).

Lesion length could not be reliably measured because of the overlying bridge. The lesion was predilated and sized with a 2.0 × 18 mm semi-compliant balloon (*Figure 4E*), and a 2.5 × 18 mm drug-eluting stent was implanted with full coverage (*Figure 5A* and *B*). Post-deployment IVUS showed underexpansion (minimum stent area 3.82 mm^2^ of 5.73 mm^2^ reference; plaque burden 33%) (*Figure 5C*); high-pressure post-dilatation with a 2.5×8 mm non-compliant balloon increased (*Figure 5D*) this to 4.83 mm^2^ of 7.51 mm^2^ reference (∼64%), with a final vessel diameter of 2.48 mm and no dissection or perforation (*Figure 5E–H*, see Supplementary material online, *Video S3*).

Aspirin 300 mg and clopidogrel 600 mg were loaded, followed by aspirin 100 mg and clopidogrel 75 mg once daily. A beta-blocker was added for the myocardial bridge; a statin was with held given the absence of atherosclerosis. He was discharged the next day. At 1-month follow-up, chest pain had markedly improved, though T-wave inversion persisted. The patient did not attend later visits; repeat left ventricular or coronary imaging was not obtained.

## Discussion

The initial angiogram showed a myocardial bridge with stenosis pronounced during systole, leading to the attribution of the stenosis to the bridge and the decision to defer PCI. One month later, symptom recurrence prompted a retrospective review of the outside angiogram and additional work-up at our centre. Coronary CTA was crucial in mapping the relationship between the pledget material and the stenotic LAD segment, indicating an external mechanical component to the compression.^6^ Potential causes include constriction by a pledget, fibrosis, a foreign-body reaction, or a combination thereof. There are documented cases of BioGlue-related coronary stenosis caused by perivascular fibrosis.^7^ Neither CTA nor IVUS can conclusively differentiate these mechanisms, and histopathology was not performed; this is a limitation in this case. Nonetheless, this highlights the importance of multimodal imaging in cases where atherosclerosis is absent and a nonconventional aetiology is suspected.

The fixed perfusion defect seen on MPI indicates an established injury or scar rather than ongoing ischaemia. This could stem from the initial trauma, the repair process, or later LAD issues.^8^ Although CMRI might have clarified the extent of scarring and myocardial viability, it was not performed due to its longer imaging time and the patient's limited cooperation.^9^ The invasive physiological assessment was not pursued due to the nearby dynamic myocardial bridge, which could affect FFR/iFR readings.^10,11^ Given this diagnostic uncertainty, the Heart Team model was central to management.^12^ Surgical revascularization was considered, but the small, distally attenuated LIMA was unlikely to reach the mid-to-distal lesion, favouring a percutaneous approach.^13^ IVUS helped rule out an atherosclerotic phenotype, size the vessel, and optimize the procedure, but PCI does not eliminate the external substrate.^14^ Ongoing forces can cause issues like underexpansion, recoil, restenosis, deformation, or fracture, so close monitoring is crucial, especially in an 18-year-old. The symptomatic response at one month is a positive early indicator, but further follow-up is necessary.

This case is limited by one-month follow-up, unconfirmed mechanism, lack of invasive physiology and CMR, and uncertainty whether stenosis developed progressively or was present since surgery and only later recognized. Nonetheless, timely imaging and personalized, multidisciplinary management led to a favourable early outcome, supporting IVUS-guided PCI as a feasible option in this rare setting.

## Patient perspective

The patient reported that his chest pain affected daily activities and caused anxiety due to prior cardiac surgery. He felt relief after the procedure, noting chest pain had resolved at follow-up. He was satisfied with the diagnosis and treatment, understanding the need for follow-up and dual antiplatelet therapy at discharge. However, he later declined further clinic visits, highlighting a common challenge in long-term follow-up of young patients.

## Lead author biography


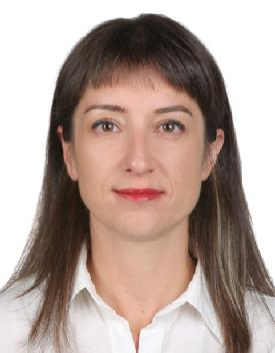
Dr. Hatice Ozdamar is a cardiologist at the Department of Cardiology, Tepecik Training and Research Hospital, Izmir, Turkiye. She has clinical and academic interests in interventional cardiology, cardiac imaging, and complex coronary interventions. She has been actively involved in multidisciplinary cardiac care and case-based research. This case represents her work at the intersection of cardiovascular imaging and interventional decision-making in rare and challenging clinical scenarios.

## Supplementary Material

ytag621_Supplementary_Data

## Data Availability

All data relevant to this case are included within the article.
